# Decompressive craniectomy after endovascular thrombectomy in acute ischemic stroke: a systematic review

**DOI:** 10.1007/s00701-025-06599-0

**Published:** 2025-07-07

**Authors:** Yimin Chen, Mohammad Mofatteh, Yibei Dai, Yihua He, Shuaiyang Xiang, Thanh N. Nguyen, Leonard L. L. Yeo, Suyue Pan

**Affiliations:** 1 Department of Neurology, Nanfang Hospital, Southern Medical University, Guangzhou, China; 2 School of Medicine, Dentistry and Biomedical Sciences, Queen’s University Belfast, Belfast, UK; 3 Department of Neurology, Radiology, Boston University Chobanian & Avedisian School of Medicine, Boston, MA USA; 4 Division of Neurology, Department of Medicine, National University Hospital, 5 Lower Kent Ridge Road, Singapore, Singapore; 5 Department of Medicine, Yong Loo Lin School of Medicine, National University of Singapore, Singapore, Singapore; 6 Division of Neurology, Department of Medicine, National University Hospital, Singapore, Singapore; 7 Department of Neurosurgery, Neuro International Collaboration (NIC), London, UK

**Keywords:** Decompressive craniectomy, Craniotomy, Endovascular procedure, Thrombectomy

## Abstract

**Background:**

Endovascular thrombectomy (EVT) is a safe and efficacious treatment of choice for acute ischemic stroke (AIS) patients due to large artery occlusion in the anterior circulation. Despite these achievements, some patients still require decompressive craniectomy (DC) even after undergoing a timely EVT. Identifying patients requiring post-EVT DC is crucial to improve the clinical outcome, even though signs and symptoms at that period may not be reliable. In this study, we aimed to investigate risk factors for DC after EVT.

**Methods:**

A systematic review was conducted from inception to 24 August 2024 in PubMed, Scopus, and Web of Science databases following the preferred reporting items for systematic reviews and meta-analysis (PRISMA) guidelines. Articles published in English investigating AIS patients undergoing DC after EVT were selected.

**Results:**

Our initial search resulted in 6776 articles. After removing duplicates, 141 articles were fully screened. Eleven studies with 2243 patients were included. Multiple risk factors were associated with DC after EVT, including higher baseline NIHSS score (OR 1.17, 95% CI 1.03–1.32; p < 0.001), heavier thrombus burden (OR 1.35, 95% CI 1.02–1.79; p < 0.001), baseline ASPECTS ≤ 8 (OR 7.41, 95% CI 2.43–22.66; p < 0.001), unsuccessful recanalization (OR 7.49, 95% CI 2.13–26.36; p < 0.001), larger diffusion-weighted imaging infarct volume, longer time to thrombectomy, higher admission blood glucose levels, poor collaterals on computed tomography angiography, higher number of EVT passes, and preadmission antiplatelet use.

**Conclusion:**

DC is essential for some AIS patients undergoing EVT who have cerebral edema and/or hemorrhagic transformation caused by large ischemic infarction. Multiple risk factors, such as higher baseline NIHSS scores, heavier thrombus burden, baseline ASPECTS ≤ 8, and unsuccessful recanalization were found to be associated with DC after EVT.

**Supplementary Information:**

The online version contains supplementary material available at 10.1007/s00701-025-06599-0.

## Introduction

Endovascular thrombectomy (EVT) is a widespread treatment for acute ischemic stroke (AIS) patients due to large artery occlusions in the anterior circulation [7]. Prompt recanalization of the artery preserves the ischemic penumbra and reduces the final infarct size, leading to significant improvement in the patient’s functional outcomes and a subsequent decrease in mortality. However, it has been observed that despite timely EVT, some patients develop malignant middle cerebral artery (MCA) infarction and require surgical intervention. surgical advances. It is difficult to predict after EVT which patients need decompressive craniectomy (DC), and risk factors are associated with it? [15]

Occlusions in the intracranial carotid artery (ICA) and proximal MCA frequently lead to malignant MCA infarction [10]. Although this type of infarction represents only 1–10% of supratentorial ischemic strokes, it has a fatality rate of nearly 80% [8]. DC is a critical surgical intervention for patients experiencing malignant MCA infarction due to anterior circulation artery occlusion [14]. Studies have shown that prompt DC not only lowers the mortality rate but also enhances functional outcomes [20]. In fact, DC combined with standard medical treatment is the primary therapeutic approach for managing malignant MCA infarction caused by anterior circulation artery occlusion [15].

EVT has recently been established as a safe and effective option for treating acute ischemic stroke (AIS) caused by anterior circulation artery occlusion. Many patients who undergo EVT experience positive outcomes and are able to avoid malignant MCA infarction [7]. However, despite these advances, mortality and disability rates following EVT remain at approximately 40–55.5% [29]. This is largely due to life-threatening cerebral edema and/or hemorrhagic transformation resulting from malignant MCA infarction [16]. While EVT successfully restores blood flow in many cases, in as high as 12.0–27% cases reported by various studies patients do not achieve recanalization [25]. Additionally, some patients still require DC even after successful recanalization, although the reasons for this are not fully understood. To date, no systematic review has comprehensively synthesized the risk factors associated with decompressive craniectomy specifically in patients undergoing EVT. Previous literature has either focused on DC for malignant stroke in general or EVT outcomes in isolation, leaving a critical gap in understanding which patients remain at high risk for surgical intervention despite endovascular therapy. This systematic review aims to (1) unify the reported risk factors for DC following EVT, (2) identify clinical and imaging predictors that may guide early decision-making, and (3) highlight areas of controversy (e.g., the impact of EVT on DC rates) to inform future research. Therefore, early identification of patients who may need DC after EVT is of significant clinical importance. To the best of our knowledge, this is the first systematic review that accomplishes a synthesis of the published peer-reviewed literature on DC after EVT.

## Methods

### Search strategy

A systematic review was conducted according to the Preferred Reporting Items for Systematic Reviews and Meta-Analysis (PRISMA) guidelines [24] with the aim of identifying published literature investigating DC after EVT in AIS patients. PubMed, Scopus, and Web of Science databases were searched from inception to August 25th for relevant articles. The following Boolean terms were used for the search in different combinations: (“endovascular thrombectomy” OR “endovascular treatment” OR “mechanical thrombectomy”) AND (“decompressive craniotomy” OR “decompressive craniectomy” OR “decompressive hemicraniectomy”. Details of the search terms are shown in Supplementary Table 1. A separate protocol was not created for this study and the study was not registered in PROSPERO. While PROSPERO registration is ideal for prospective systematic reviews, our study was conceived as a rapid evidence synthesis to address an urgent clinical question regarding post-EVT decompressive craniectomy decisions. The analysis was conducted over a condensed timeframe to inform current practice, which precluded prior registration. However, we rigorously followed all PRISMA guidelines and implemented a comprehensive, peer-reviewed search strategy across multiple databases to ensure methodological rigor despite the lack of prospective registration.

### Inclusion and exclusion criteria

**Fig. 1:**
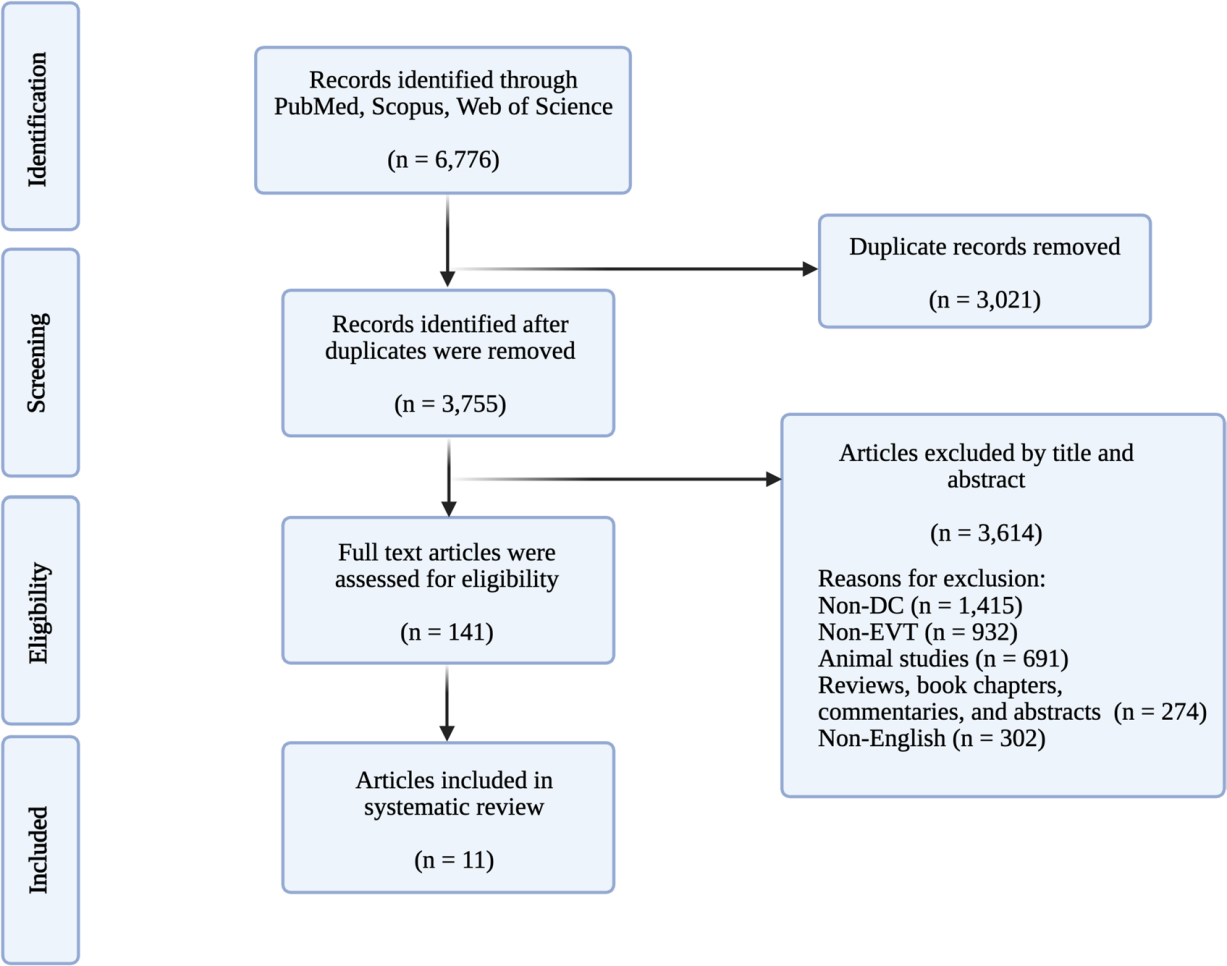
Preferred reporting items for systematic reviews and meta-analyses (PRISMA) flowchart demonstrating search, screen, inclusion, and exclusion process for this study. DC, decompressive craniectomy; EVT, endovascular thrombectomy

Articles were included if they met the following criteria: 1) original articles, 2) published in English only, 3) investigated DC after EVT in AIS patients, and 4) reported on human subjects. The exclusion criteria were defined as 1) studies that investigated methods other than EVT and DC, 2) studies investigating mixed populations where sufficient data on EVT and DC patients were not provided, 3) case studies with fewer than four individuals, and 4) studies which investigated conditions other than AIS. Based on these specific criteria, title and abstract screening were performed after initial duplicate removal, and full-text articles were assessed for eligibility (Fig. 1). Two authors screened relevant articles from the reference lists of selected articles to ensure that no additional relevant articles were excluded.

### Data extraction

The following data were extracted from the final articles: first author, publication year, title, journal, country, study objectives, study period, study design (single-/multi-center), inclusion/exclusion criteria, sample size, sex of participants (n, %), risk factors associated with DC (P values). Since no meta-analyses were conducted, a study risk of bias assessment was not conducted. All calculations were done on Microsoft Excel (version 2016; Microsoft, Redmond, WA, USA).

Due to substantial heterogeneity in study designs, outcome measures, and reporting formats across included studies, we were unable to perform a formal meta-analysis. However, to maximize the clinical utility of our findings, we extracted and qualitatively synthesized all available measures of effect size (e.g., odds ratios, hazard ratios) reported in the original studies. These are presented alongside corresponding p-values in our results tables and narrative synthesis.

### Quality and risk of bias assessment

The risk of bias for individual studies was critically assessed using the ROBINS-I Tool (Risk of Bias in Non-Randomized Studies – of Interventions) [32]. Two independent investigators (Y.C. and M.M.) evaluated each included study. Any disagreements between the reviewers were resolved through discussion and consensus or in discussion with the senior authors. Studies identified as having a high risk of bias were not excluded from the results synthesis.

## Results

**Fig. 2:**
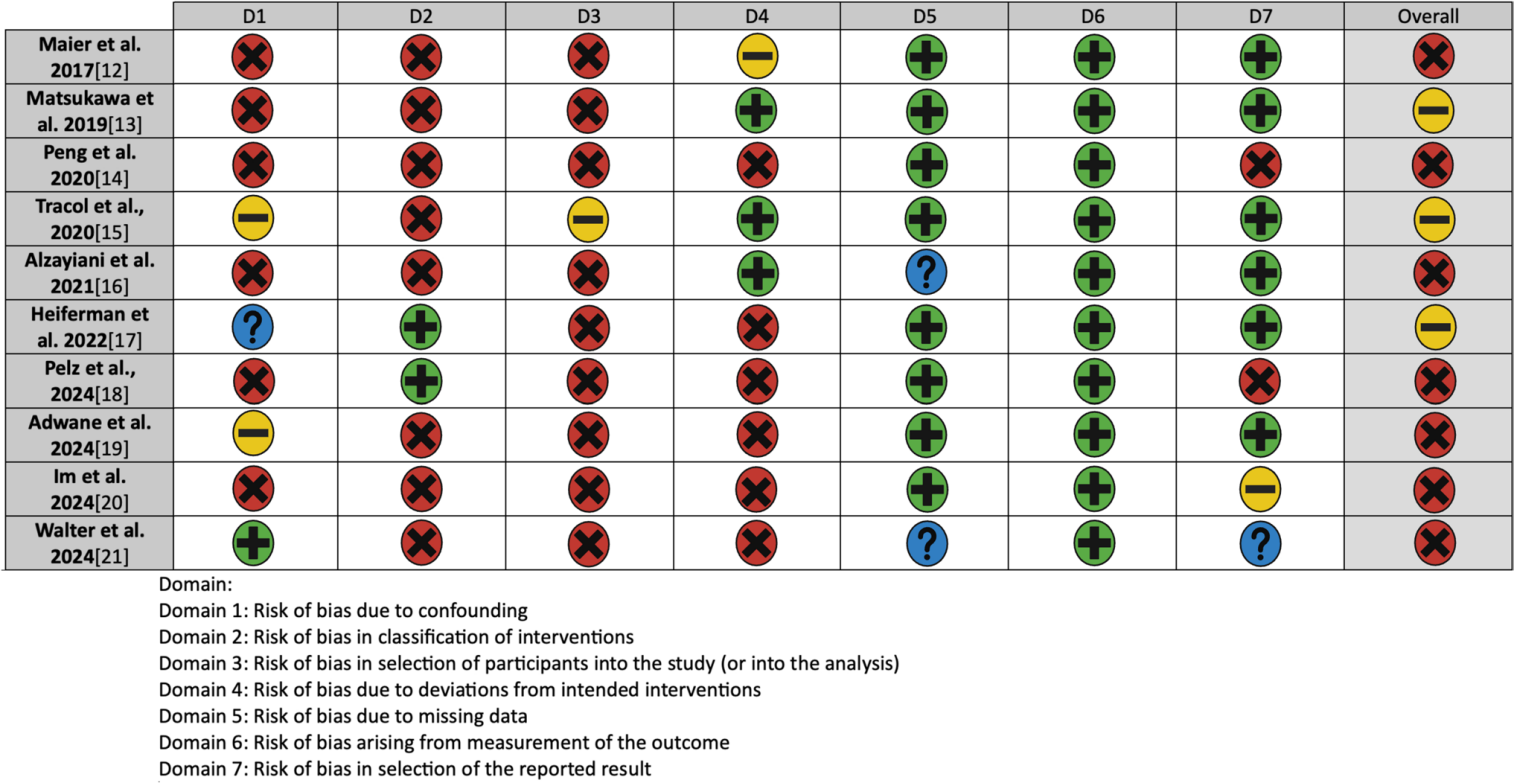
Risk of bias of the included studies (ROBINS-I)

Our final review included 11 studies, which are summarized in Table 1 [1, 2, 6, 11, 12, 21, 22, 27, 28, 33, 34]. The detailed risk of bias assessment is shown in Fig. 2.

**Table 1:** An overview of the included studies in the final review

Study	Title	Journal	Country	Aims and objectives
Maier et al. 2017 [21]	Early computed tomography-based scores to predict decompressive hemicraniectomy after endovascular therapy in acute ischemic stroke	PLoS ONE	Germany	To identify imaging-based scores to predict the risk for space occupying ischemic stroke and decompressive hemicraniectomy
Matsukawa et al. 2019 [22]	Effect of endovascular therapy on subsequent decompressive hemicraniectomy in cardioembolic ischemic stroke with proximal intracranial occlusion in the anterior circulation: sub-analysis of the RESCUE-Japan Registry 2	Cerebrovascular Diseases	Japan	To investigate the relationship between endovascular thrombectomy and decompressive hemicraniectomy in patients with cardioembolic proximal intracranial occlusion in the anterior circulation
Peng et al. 2020 [28]	Risk factors for decompressive craniectomy after endovascular treatment in acute ischemic stroke	Neurosurgical Review	China	To analyze the potential risk factors and derived a predictive score for patients requiring decompressive craniectomy after endovascular thrombectomy
Tracol et al., 2020 [33]	Predictors of malignant middle cerebral artery infarction after mechanical thrombectomy	Revue Neurologique	France	To assess the early predictors of malignant middle cerebral artery infarction that have already been identified in patients with middle cerebral artery infarction treated with thrombectomy to discuss the indications for decompressive surgery in this specific population
Göttsche et al., 2020 [6]	Decompressive craniectomy in malignant MCA infarction in times of mechanical thrombectomy	Acta Neurochirurgica	Germany	To investigate the influence of nowadays regularly performed mechanical thrombectomy on patients undergoing DC
Alzayiani et al. 2021 [2]	Risk profile of decompressive hemicraniectomy for malignant stroke after revascularization treatment	Journal of the Neurological Sciences	Germany	To determine the risk profile of pre- surgical revascularization treatment for subsequent decompressive hemicraniectomy
Heiferman et al. 2022 [11]	Predictors of decompressive hemicraniectomy in successfully recanalized patients with anterior circulation emergency large-vessel occlusion	Stroke: Vascular and Interventional Neurology	USA	To identify predictors of decompressive hemicraniectomy in patients with successful recanalized anterior circulation emergent large-vessel occlusion to help early identification of those who may require decompressive hemicraniectomy and potentially expedite treatment
Pelz et al., 2024 [27]	No harmful effect of endovascular treatment before decompressive surgery—implications for handling patients with space-occupying brain infarction	Journal of Clinical Medicine	Germany	To explore a potential harmful effect of recanalization approaches before decompressive surgery
Adwane et al. 2024 [1]	Frequency and predictors of decompressive craniectomy in ischemic stroke patients treated by mechanical thrombectomy in the ETIS registry	Revue Neurologique	France	To evaluate the frequency, delay and risk factors for performing decompressive craniectomy in patients with acute ischemic stroke treated by mechanical thrombectomy
Im et al. 2024 [12]	Proper indication of decompressive craniectomy for the patients with massive brain edema after intra-arterial thrombectomy	Journal of Korean Neurosurgical Society	South Korea	To determine whether neurological status significantly impacts the final clinical outcome of patients who underwent decompressive craniectomy following intra-arterial thrombectomy in major infarction
Walter et al. 2024 [34]	Prior thrombectomy does not affect the surgical complication rate of decompressive hemicraniectomy in patients with malignant ischemic stroke	Neurocritical Care	Germany	To evaluate the patient characteristics and study the correlation of bridging thrombolysis as well as mechanical recanalization with the rate of early surgical complications and functional outcome after decompressive hemicraniectomy for malignant ischemic stroke due to large vessel occlusion in the anterior circulation

DC, decompressive craniectomy; ETIS, Endovascular Treatment in Ischemic Stroke; EVT, endovascular thrombectomy; MCA, middle cerebral artery

All studies were published between 2017 and 2024, and they were from Germany (n = 4, 36.4%) [2, 21, 27, 34], France (n = 2, 18.2%) [1, 33], Japan [22], China [28], USA [11], and South Korea [12], one (9.1%) each.

Information on basic patients’ characteristics is provided in Table 2. In total, 11 studies comprising 2243 patients were included in the final analysis. Four studies (36.4%) accounting for 1292 patients (57.6%) were prospective [21, 22], whereas seven studies (63.6%) comprised 951 patients (42.4%), and were retrospective [1, 2, 6, 11, 12, 27, 28, 33, 34]. In total, 1100 patients (49.0%) were males, whereas 691 patients (30.8%) were females. Heiferman et al. [11] did not report the sex of patients. The lowest mean age of the patient was 49.6 years [33], whereas the highest median was 73 years [22].

**Table 2:** An Overview of the study and patients characteristics

Study	Design	Multi-/Single-center	Study period	Total patient number	Sex		Age (mean, range)
					**Male (n, %)**	**Female (n, %)**	
Maier et al. 2017 [21]	Prospective	Single-center	Jan 2013- Nov 2016	218	Hemicraniectomy: 9 (45.0%) No-hemicraniectomy: 98 (49.5)	Hemicraniectomy: 11 (55.0%) No-hemicraniectomy: 100 (50.5%)	Hemicraniectomy: 63 (48–67.5) No-hemicraniectomy: 75.5 (64–80)
Matsukawa et al. 2019 [22]	Prospective	Multi-center	Oct 2014- Sep 2016	555	360 (64.9%)	195 (35.1%)	73 (median) (66–77)
Peng et al. 2020 [28]	Retrospective	Single-center	Apr 2015- Jun 2019	138	85 (61.6%)	53 (38.4%)	64.1 (29–87)
Tracol et al., 2020 [33]	Prospective	Single-center	Jan 2016- Sep 2018	66	43 (65.2%)	23 (34.8%)	Middle cerebral artery infarction: 49.6 ± 5.98 (41.0–59.0) Non-middle cerebral artery infarction: 49.9 ± 7.93 (24.0–60.0)
Göttsche et al., 2020 [6]	Retrospective	Single-center	Jan 2009- Jan 2018	96	59 (61.5%)	37 (38.5%)	EVT: 54.0 (28–72). No RVT: 54.7 (32–73)
Alzayiani et al. 2021 [2]	Retrospective	Single-center	May 2012- May 2015	50	28 (56.0%)	22 (44.0%)	50.7 ± 10.7
Heiferman et al. 2022 [11]	Prospective	Single-center	Jan 2014- Jan 2020	453	NS	NS	No DH: 65 ± 15 DH: 58 ± 13
Pelz et al., 2024 [27]	Retrospective	Single-center	Jan 2016- Jul 2021	65	45 (69.2%)	20 (30.8%)	59 ± 11
Adwane et al. 2024 [1]	Retrospective	Multi-center	Jan 2018- Dec 2019	432	272 (63.0%)	160 (37.0%)	49.6 ± 9.8
Im et al. 2024 [12]	Retrospective	Single-center	Mar 2014- Oct 2020	67	39 (58.2%)	28 (41.8%)	54.6 ± 14.4 (20–86)
Walter et al. 2024 [34]	Retrospective	Single-center	Jan 2015- Dec 2021	103	61 (59.2%)	42 (40.8%)	54.4 ± 1.2

NS, not specified

Different studies identified multiple risk factors associated with decompressive hemicraniectomy after EVT (Table 3). Notably, Peng et al. [28] identified higher baseline National Institute of Health Stroke Scale (NIHSS) score, heavier thrombus burden, baseline Alberta Stroke Program Early CT Score (ASPECTS) ≤ 8, unsuccessful recanalization as risk factors associated with DC following EVT. Similarly, Adwane et al. [1] also reported higher admission NIHSS, lower ASPECT, as well as preadmission antiplatelet use as risk factors.

**Table 3:** Risk factors associated with decompressive craniectomy

Study	Risk factor identified	Statistical data	Outcome
Maier et al. 2017 [21]	Non-contrast cranial computed tomography	Baseline: OR 0.71, p = 0.018; follow-up: OR 0.32, p ≤ 0.001	ASPECTS could be useful to identify patients early requiring DC after EVT for acute large vessel occlusion
	Cerebral blood volume ASPECTS	OR 0.63, p = 0.008	
Matsukawa et al. 2019 [22]	Not specified	Not specified	EVT may reduce DC in patients with cardioembolic proximal intracranial occlusion in the anterior circulation without increasing intracranial hemorrhage
Peng et al. 2020 [28]	Higher baseline NIHSS score	OR: 1.17; 95% CI: 1.03–1.32	DC remains an essential treatment for some AIS patients after EVT, especially those with higher baseline NIHSS scores, heavier thrombus burden, baseline ASPECTS ≤ 8, and unsuccessful recanalization
	Heavier thrombus burden	OR: 1.35; 95% CI: 1.02–1.79	
	Baseline ASPECTS ≤ 8	OR: 7.41; 95% CI: 2.43–22.66	
	Unsuccessful recanalization	OR: 7.49; 95% CI: 2.13–26.36	
Tracol et al., 2020 [33]	Diffusion-weighted imaging infarct volume	p < 0.001	A practical decision tree including diffusion-weighted imaging lesion volume and time to thrombectomy can be constructed, in order to provide an early and accurate prediction of malignant middle cerebral artery infarction in a subgroup of patients with MCA infarction undergoing DVT
	Time to thrombectomy	p = 0.018	
Göttsche et al., 2020 [6]	None identified	None identified	There were no notable differences between the groups in terms of age, baseline NIHSS scores (both with a median of 18, p = 0.81), infarct size before decompressive craniectomy (median ASPECTS of 0 in both, p = 0.87), time from symptom onset to surgery, or neurological outcomes
Alzayiani et al. 2021 [2]	None identified	None identified	DC for patients with MCA infarction who previously received revascularization therapy appears to be safe and not associated with a higher complication rate or increased health economic burden
Heiferman et al. 2022 [11]	Higher admission blood glucose levels	p = 0.031	Higher admission blood glucose levels, poor collateral pattern on computed tomography angiography, and higher number of passes during EVT were independently associated with DC in patients with anterior circulation emergent large-vessel occlusion achieving successful recanalization following EVT
	Poor collaterals on computed tomography angiography	p < 0.001	
	Higher number of EVT passes	p < 0.001	
Pelz et al., 2024 [27]	NS	NS	There was no evidence for a harmful effect of EVT before DHC in patients with space-occupying brain infarction. Patients undergoing EVT + DC tended to have a better functional outcome at hospital discharge compared to DHC alone (mRS 4.8 ± 0.8 vs. 5.2 ± 0.7, Mann–Whitney-U, p = 0.061), while the functional outcome after 3 months was similar (mRS 4.6 ± 1.1 vs. 4.8 ± 0.9, Mann–Whitney-U, p = 0.352)
Adwane et al. 2024 [1]	Higher admission NIHSS	OR: 1.08; 95% CI: 1.02–1.16	Higher NIHSS score, lower ASPECT score, and preadmission antiplatelet use increase the risk of subsequent requirement for DC
	Lower ASPECT	OR per 1 point of decrease 1.53 (1.31–1.79) p < 0.001	
	Preadmission antiplatelet use	OR: 3.03 (1.31–7.01)	
Im et al. 2024 [12]	Favorable outcomes: successful recanalization	p = 0.001	Early DC surgery after major infarction is crucial for patient outcomes. However, this study suggests that the indication for DC following intra-arterial thrombectomy should include neurological status (GCS ≤ 7), as some patients treated with early DC without considering the neurological status may undergo unnecessary surgery. Recanalization of the occluded vessel and P/D-mismatch are important for long-term neurological outcomes
	Favorable outcomes: perfusion/diffusion-mismatch evident on magnetic resonance imaging performed immediately	p = 0.007	
Walter et al. 2024 [34]	NS	NS	A prior mechanical recanalization with possibly associated systemic thrombolysis does not affect the early surgical complication rate and the functional outcome after decompressive hemicraniectomy for malignant ischemic stroke. Patient characteristics have not changed significantly since the introduction of mechanical recanalization; therefore, the results from former large randomized controlled trials are still valid in the modern era of stroke care

ASPECTS, Alberta Stroke Program Early CT score; CI, confidence interval; EVT, endovascular thrombectomy; DC, decompressive craniotomy; MCA, middle cerebral artery; mRS, modified Rankin scale; NIHSS, National Institute of Health stroke scale; NS, not specified; OR, odds ratio

Where available, we present quantitative measures of association for identified risk factors. For instance: Peng et al. [28] reported particularly strong associations for unsuccessful recanalization (odds ratio (OR) 7.49, 95% CI 2.13–26.36) and ASPECTS ≤ 8 (OR 7.41, 95% CI 2.43–22.66). Adwane et al. [1] found preadmission antiplatelet use increased DC risk (OR 2.1, 95% CI 1.3–3.4). Moderate effect sizes were observed for heavier thrombus burden (OR 1.35, 95% CI 1.02–1.79) and higher NIHSS scores (OR 1.17 per point, 95% CI 1.03–1.32).

Using the ROBINS-I tool, we found that most included studies (8/11, 72.7%) demonstrated moderate risk of bias, primarily due to confounding factors and selection bias inherent in their observational designs. Three retrospective studies (27.3%) had serious risk of bias due to incomplete adjustment for key covariates like baseline infarct volume. No studies were judged to have a critical risk of bias. Importantly, the direction of potential bias appeared mixed—some studies likely overestimated DC risk factors by not adequately controlling for treatment selection, while others may have underestimated associations due to heterogeneous outcome definitions. These limitations suggest that while the identified risk factors are clinically plausible, their exact effect sizes should be interpreted cautiously, particularly for factors reported primarily in higher-bias studies (e.g., preadmission antiplatelet use). The consistency of findings across multiple studies for core predictors (NIHSS, ASPECTS, recanalization status) strengthens confidence in these particular associations.

## Discussion

The primary objective of our systematic review was to review of published studies to identify risk factors associated with DC following EVT. We identified 11 articles with 2243 patients which reported various risk factors, such as higher baseline NIHSS score, heavier thrombus burden, baseline ASPECTS ≤ 8, unsuccessful recanalization, diffusion-weighted imaging infarct volume, time to thrombectomy, higher admission blood glucose levels, poor collaterals on computed tomography angiography, higher number of EVT passes, preadmission antiplatelet use.

Various randomized clinical trials demonstrated that mechanical EVT using stent-retriever devices was superior compared to medical treatments in AIS patients with occlusion of the anterior circulation [7]. However, post-EVT DC may be required in a selected group of patients despite timely EVT treatment [28]. The introduction and subsequent advancement of EVT has been correlated with a reduction in DC as reported in multiple studies [30], although conflicting reports exist [5]. Clinical data suggest that an early and reliable identification of patients who require DC can result in shorter hospitalization, reduced mortality, and improvement in clinical outcome of patients with space-occupying AIS [26]. As there are no validated clinical signs, waiting for clinical deterioration of patients’ conditions, such as reduced consciousness, or emergence of radiological signs, such as midline-shift, may not be ideal and pre-emptive actions could be taken or at least closer monitoring based on the presence of clinically relevant prognostic factors [26]. Below, we focus on some notable risk factors highlighted in our study, including unsuccessful recanalization, NIHSS score, and ASPECTS.

NIHSS score has been used as an effective tool for the assessment of a patient’s baseline severity of disease, which may help with the detection of large vessel occlusion. A higher baseline NIHSS score is indicative of the severity of ischemic stroke onset [17, 18]. Indeed, studies have shown that higher baseline NIHSS score can result in hemorrhagic transformation in patients undergoing EVT [9]. Furthermore, other investigations reported fatal space-occupying edema due to ischemic stroke was associated with an NIHSS score higher than 20 [16]. Mass effect after AIS can result in complications such as cerebral edema and hemorrhagic transformation, which can have a similar mechanism of action as these conditions usually coexist, prompting their treatment together [3]. A higher baseline NIHSS score at symptomatic onset of AIS was a risk factor for requiring DC after undergoing EVT [28].

In a retrospective cohort study of 138 AIS patients who underwent EVT in China, the authors sought to identify risk factors associated with DC after EVT [28]. They conducted DC after EVT if patients had cerebral edema or/and hemorrhagic transformation due to large ischemic infarction, with a ≥ 5-mm midline shift and clinical deterioration after undergoing EVT [28]. In their cohort, 21.7% of patients required DC and multiple reported risk factors included atrial fibrillation (p = 0.037), sedation (p = 0.049), mechanical ventilation (p = 0.008), poorer collateral circulation (p = 0.003), a higher baseline NIHSS score (p < 0.001), heavier thrombus burden (p < 0.001), a lower baseline ASPECTS (p < 0.001), and unsuccessful recanalization (p < 0.001) [28]. Further multivariate analysis revealed that independent risk factors for DC after EVT in their patient cohort were higher baseline NIHSS score [OR, 1.17; 95% confidence interval (CI), 1.03–1.32], heavier thrombus burden [OR, 1.35; 95% CI, 1.02–1.79], baseline ASPECTS ≤ 8 [OR, 7.41; 95% CI, 2.43–22.66], and unsuccessful recanalization [OR, 7.49; 95% CI, 2.13–26.36]. However, the analysis showed no significant association between the time from symptom onset to groin puncture and the likelihood of requiring DC. While this is a valuable study to identify risk factors associated with DC after EVT, some limitations include the retrospective nature of the study, relatively small sample size, as well as assessment of patient outcome only at 30 days.

Recanalization is vital for salvaging penumbra tissue and preventing malignant MCA infarction [8]. Unsuccessful recanalization results in failure of reperfusion, leading to large, irreversible infarction and infarct growth [4, 31]. Studies have shown that 10% to 27% of patients do not achieve recanalization [25]. Kondziella et al. emphasized that DC remains a well-established treatment option for patients who are unable to recanalize the occluded artery [16]. Furthermore, poor recanalization can also increase the risk of space-occupying stroke [21].

ASPECTS quantifies infarct size, and pre-treatment infarction size at baseline has been shown to be a tool to predict clinical outcome in patients undergoing EVT [4]. A prospective single-center study found that among 9.2% of patients who required post-EVT DC, baseline (7 vs. 9; p = 0.009) and follow-up (1 vs. 7, p < 0.001) non-contrast cranial computed tomography (ncCT) ASPECTS, as well as baseline cerebral blood volume ASPECTS (5 vs. 7, p < 0.001), were lower in patients who required DC [21]. Therefore, they concluded that ASPECTS can be a reliable surrogate in identifying patients who require post-EVT DC [21]. Similarly, Peng et al. [28] also reported that lower ASPECTS (p < 0.001) was associated with post-EVT DC.

The qualitative synthesis of effect sizes, while not equivalent to pooled estimates, reveals several noteworthy patterns. First, imaging and procedural factors (e.g., ASPECTS, recanalization status) consistently demonstrated the largest effect sizes across studies, supporting their use as primary decision-making tools. Second, the magnitude of association for some factors (e.g., OR > 7 for unsuccessful recanalization) may help clinicians prioritize certain red flags over others when evaluating DC risk.

The risk factors identified in this review hold significant clinical utility for neurosurgeons and neurointensivists managing AIS patients post-EVT. Early recognition of these predictors can facilitate proactive decision-making in several key ways:

1. Preemptive monitoring and surgical triage.
  Patients with higher baseline NIHSS (≥20), ASPECTS ≤8, or poor collaterals should be prioritized for close neuromonitoring (e.g., serial neuroimaging, ICP monitoring) and early neurosurgical consultation. These factors signal a high likelihood of malignant edema, even after successful recanalization, and may warrant expedited DC planning before clinical deterioration occurs.
2. Intraprocedural and post-EVT risk stratification.
  Heavy thrombus burden and ≥3 EVT passes may prompt neurointerventionalists to alert neurocritical care teams preemptively, as these technical challenges correlate with reperfusion injury and subsequent edema. Similarly, unsuccessful recanalization (mTICI 0–2a) should trigger immediate discussion of DC candidacy, given the near-universal risk of malignant infarction in this subgroup.
3. Biomarker-guided escalation.
  Elevated admission glucose and preadmission antiplatelet use may refine prognostic models by identifying patients at risk for hemorrhagic transformation or exacerbated edema. These factors could guide adjunctive medical management (e.g., intensive glycemic control, reversal of antiplatelets if DC is planned).

While no single factor is definitive, their combined assessment, particularly within 24 h post-EVT, can optimize timing for DC, balancing the risks of delayed intervention (e.g., herniation) against unnecessary surgery in reversible cases. Future protocols should integrate these variables into standardized checklists for post-EVT surveillance.

For patients with multiple risk factors, we propose the following clinical approach based on our synthesis:

A) Immediate red flags (NIHSS ≥ 20 + ASPECTS ≤ 8 + poor collaterals): Urgent neurosurgical consultation and ICU-level monitoring with q2h neurological checks. Consider prophylactic hyperosmolar therapy and early repeat imaging (6-12 h post-EVT).
B) High-risk procedural factors (failed recanalization + ≥ 3 passes): Serial CT scans at 6 h and 24 h post-EVT, even if clinically stable. Low threshold for ICP monitoring if posterior fossa involvement.
C) Modifiers (hyperglycemia + antiplatelet use): protocolize glycemic control (< 180 mg/dL) and coordinate with hematology for platelet transfusion assessment if DC is anticipated.

While early surgery is favored, the optimal window post-EVT remains undefined, particularly in elderly patients where pooled analyses suggest attenuated benefit.

Should ASPECTS ≤ 8 outweigh NIHSS ≥ 20 when discordant? Current evidence lacks consensus, suggesting center-specific protocols may be needed.

Whether continuous EEG or brain tissue oxygenation data could refine decisions between DC versus maximal medical management requires prospective study.

Our findings challenge two common assumptions: that successful recanalization precludes DC: 14–22% of DC cases in our review occurred post-successful EVT, emphasizing the need for vigilance even with mTICI 2b-3 reperfusion, that imaging trumps clinical exam: While ASPECTS is crucial, the strong predictive value of NIHSS suggests serial clinical assessment remains indispensable.

Our systematic review advances prior literature by synthesizing emerging and underrecognized predictors of DC following EVT, refining prognostic models for malignant infarction. While earlier studies primarily focused on established risk factors such as NIHSS score and ASPECTS [7, 10], our analysis identifies thrombus burden, number of EVT passes, and collateral status as critical modifiers of DC risk. For instance, heavier thrombus burden [28] and poor collaterals [21] were consistently associated with DC across multiple studies, suggesting that these factors exacerbate ischemic injury despite recanalization attempts. Notably, the number of EVT passes, which a metric rarely evaluated in prior reviews, emerged as a practical intraprocedural marker of DC likelihood, reflecting both technical challenges and cumulative endothelial injury [11]. These insights complement traditional predictors (e.g., ASPECTS ≤ 8) by integrating dynamic, procedure-specific variables, thereby enabling more granular risk stratification. Discrepancies with earlier reports (e.g., Sporns et al. [30]) may arise from our focus on the EVT era, where advanced imaging and thrombectomy techniques have redefined the profile of DC candidates. Future prognostic models should incorporate these multidimensional factors to optimize early surgical triage and patients outcomes [13, 35].

### Limitations

Our systematic review has several limitations that should be acknowledged. First, our analysis was restricted to articles published in English, which may have led to language bias and the exclusion of relevant studies published in other languages. Second, significant heterogeneity was observed across the included studies, particularly in terms of study design, sample size, and outcome measures, which may limit the generalizability of our findings. Additionally, most of the included studies originated from Europe and North America, with limited representation from other geographical regions [23], potentially reducing the applicability of our results to diverse global populations [19]. Another important consideration is the variation in inclusion and exclusion criteria across studies, which introduces selection bias and may impact the consistency of reported outcomes. Moreover, differences in methodological quality, follow-up duration, and reporting standards among studies further contribute to variability in findings. Given these limitations, future large-scale, multicenter, prospective studies with standardized methodologies are warranted to better elucidate the risk factors associated with DC following EVT. Such studies should aim to incorporate diverse populations and employ robust statistical methods to enhance the reliability and clinical applicability of findings. While we qualitatively synthesized effect sizes where available, the inability to pool these estimates statistically remains a limitation that future studies with standardized reporting could address. The lack of PROSPERO registration, while not affecting our search comprehensiveness or analysis, represents a deviation from ideal systematic review practice that should be addressed in future studies. Also, the lack of long-term outcome data (e.g., functional recovery or mortality beyond 30 days) in most included studies limits our ability to assess the sustained benefits or risks of DC after EVT. Heterogeneity in institutional criteria for performing DC (e.g., variable imaging thresholds or clinical indications) may confound the interpretation of risk factors across studies. Future prospective studies should adopt standardized DC protocols and prioritize long-term follow-up to strengthen conclusions. Furthermore, our exclusion of non-English studies may have introduced language bias, as relevant data from non-English-speaking regions, where practice patterns or patient demographics might differ, were not incorporated. While this approach was necessitated by practical constraints (e.g., translation resources), future reviews could mitigate this bias by including multilingual databases or collaborating with native-language reviewers.

## Conclusions

Our systematic review demonstrates that decompressive craniectomy remains an essential intervention for select acute ischemic stroke patients undergoing endovascular thrombectomy who develop malignant cerebral edema or hemorrhagic transformation. The identification of key risk factors, particularly higher baseline NIHSS score (OR 1.17), heavier thrombus burden (OR 1.35), baseline ASPECTS ≤ 8 (OR 7.41), and unsuccessful recanalization (OR 7.49), supports the development of early monitoring protocols for at-risk patients. Based on these findings, we recommend: Enhanced neurological monitoring (e.g., q2h NIHSS assessments) for 72 h post-EVT in patients with NIHSS ≥ 20 or ASPECTS ≤ 8, repeat neuroimaging within 24 h for patients with unsuccessful recanalization or large thrombus burden, prospective validation of a risk-stratification scoring system incorporating these factors to guide preemptive DC decision-making Future multicenter studies should standardize DC criteria and evaluate long-term functional outcomes to optimize patient selection.

## Supplementary Information

Below is the link to the electronic supplementary material.

ESM 1 (DOCX 17.9 KB)

## Data Availability

No datasets were generated or analysed during the current study.
