# Supplementary material for: Decompressive craniectomy after endovascular thrombectomy in acute ischemic stroke: a systematic review
Source: Acta Neurochir (Wien). 2025 Jul 7;167(1):185. doi: 10.1007/s00701-025-06599-0 (PMC12234614; doi:10.1007/s00701-025-06599-0)
Supplement: Supplementary file 1 — (DOCX 17.9 KB) [file 701_2025_6599_MOESM1_ESM.docx]

**Supplementary Table 1**. An Overview of Search Terms Used for Each Database

| **Database** | **Search results** | **Search terms** |
| --- | --- | --- |
| PubMed | 3920 | ((("Endovascular"[All Fields] AND ("thrombectomy"[MeSH Terms] OR "thrombectomy"[All Fields] OR "thrombectomies"[All Fields])) OR ("Endovascular"[All Fields] AND ("therapeutics"[MeSH Terms] OR "therapeutics"[All Fields] OR "treatments"[All Fields] OR "therapy"[MeSH Subheading] OR "therapy"[All Fields] OR "treatment"[All Fields] OR "treatment s"[All Fields])) OR (("mechanical"[All Fields] OR "mechanically"[All Fields] OR "mechanicals"[All Fields] OR "mechanics"[MeSH Terms] OR "mechanics"[All Fields] OR "mechanic"[All Fields]) AND ("thrombectomy"[MeSH Terms] OR "thrombectomy"[All Fields] OR "thrombectomies"[All Fields]))) AND ("decompressive craniectomy"[MeSH Terms] OR ("decompressive"[All Fields] AND "craniectomy"[All Fields]) OR "decompressive craniectomy"[All Fields] OR ("decompressive"[All Fields] AND "craniotomy"[All Fields]) OR "decompressive craniotomy"[All Fields])) OR ("decompressive craniectomy"[MeSH Terms] OR ("decompressive"[All Fields] AND "craniectomy"[All Fields]) OR "decompressive craniectomy"[All Fields]) OR (("decompress"[All Fields] OR "decompressed"[All Fields] OR "decompresses"[All Fields] OR "decompressing"[All Fields] OR "decompression"[MeSH Terms] OR "decompression"[All Fields] OR "decompressions"[All Fields] OR "decompressive"[All Fields]) AND ("hemicraniectomies"[All Fields] OR "hemicraniectomy"[All Fields])) |
| Scopus | 869 | ALL ( ( ( endovascular AND thrombectomy OR endovascular AND treatment OR mechanical AND thrombectomy OR decompressive AND craniotomy OR decompressive AND craniectomy OR decompressive AND hemicraniectomy ) ) ) |
| Web of Science | 1987 | ALL=((endovascular OR thrombectomy OR treatment OR mechanical) AND (decompressive craniotomy OR decompressive craniectomy OR decompressive hemicraniectomy)) |
